# Adolescent nutritional awareness and use of food labels: Results from the national nutrition health and examination survey

**DOI:** 10.1186/1471-2431-12-55

**Published:** 2012-05-28

**Authors:** Janet M Wojcicki, Melvin B Heyman

**Affiliations:** 1 Department of Pediatrics, University of California, 500 Parnassus Avenue MU4E SF, San Francisco, CA 94134-0136, USA

## Abstract

**Background:**

Awareness of federal nutrition programs and use of the nutrition facts label are associated with reduced risk for obesity and increased intake of fruits and vegetables. Relationships between nutrition programs, use of food labels and risk for overweight and obesity have rarely been evaluated in adolescents.

**Methods:**

Using the National Health and Nutrition Examination Survey from 2005–6, we evaluated the frequency of nutrition awareness of USDA and CDC nutrition programs and use of food labels in adolescents. Risk for overweight (BMI ≥ 85-94th percentile) and obesity (BMI ≥ 95th percentile) was assessed in relation to nutrition awareness and label reading.

**Results:**

Most adolescents (92.4%) were aware of the Food Guide Pyramid. Fewer (43.5%) were aware of the 5-A-Day Program, and even less (29.3%) were aware of the Dietary Guidelines for Americans. Less than 25% of adolescents decided which foods to purchase by reading material on the nutrition facts label. There were significant racial and ethnic differences in awareness of federal nutrition programs with Mexican-Americans having the lowest levels of awareness of the US Dietary Guidelines for Americans and the Food Guide Pyramid in comparison with other groups. Non-Hispanic whites had higher and African-American adolescents had lower frequencies of reading fat information on the nutrition label in comparison to Mexican-American and other Hispanics. Awareness of other nutrition programs or of other information on the nutrition facts label was not associated with increased or decreased risk for overweight or obesity.

**Conclusions:**

Use of the nutrition facts panel information is low among US adolescents. Additionally, less than half of adolescents are aware of federal nutrition programs including the Dietary Guidelines for Americans. Future studies should evaluate avenues to make nutrition information more accessible to young Americans.

## Background

Uniform nutrition labels were introduced in the US in 1994 as part of the Nutrition Labeling and Education Act (NLEA) in an attempt to provide more information to US consumers to improve eating practices. Use of the nutrition facts label has been associated with the reduction of fat and overall energy intake in experimental studies [1,2]. Knowledge about the nutrition and fat content of food predicts overall dietary intake in lab-based studies [3,4].

US federal agencies also have a number of programs and information sources where residents can get nutrition information and guidance, ideally improving the dietary intake of Americans. The US Department of Agriculture (USDA) and the US Department of Health and Human Services compiled the Dietary Guidelines for Americans in 2010, which provides dietary recommendations [5]. The USDA’s Center for Nutrition Policy and Promotion designed The Food Guide Pyramid, which provides an outline of how to structure daily food choices based on the Dietary Guidelines for Americans, an attempt to help structure daily food choice for Americans [6]. The Food Guide Pyramid has recently been replaced by MyPlate, also designed to help Americans structure daily food choices (USDA) [7]. Another program is the 5-A-Day for Better Health Program now called the Fruits and Veggie-More Matters (initially developed by the National Cancer Institute in collaboration with the Produce for Better Health Foundation and taken over by the CDC in 2005), which was designed to encourage fruit and vegetable consumption, including the provision of recipes and tips to promote fruit and vegetable consumption [8].

The efficacy of these federal programs and guidance to improve the nutritional health of Americans has not been extensively evaluated. Evidence suggests that knowledge of the Dietary Guidelines for Americans has been associated with increased likelihood of meeting dietary guidelines for dairy, protein, and intakes of fruits and vegetables [9]. However, little is known about the frequency of nutrition facts label reading or knowledge of federal programs and health outcomes in at-risk populations such as adolescents who are beginning to make food purchases for themselves. Furthermore, little information is available regarding differences based on race or ethnicity.

US adolescents are an important group to target for nutrition interventions. In 2007–8, adolescents were reported to have an unacceptably high prevalence of obesity (34.2% overweight [body mass index (BMI) ≥85^th^ percentile] and 18.1% obese [BMI ≥ 95^th^ percentile]), with an even higher prevalence in African-American and Latino populations [10]. The obesity epidemic has stimulated interest to improve the nutrition education of teenagers. The Let’s Move Obesity Prevention Campaign spearheaded by First Lady Michelle Obama stresses the need to make nutrition facts labels easier to use for consumers and the need for better dissemination of information provided in the Food Guide Pyramid and the Dietary Guidelines for Americans 2010 to help address the obesity epidemic [11]. We used the National Health and Nutrition Examination Survey (NHANES) 2005–6 to determine frequency of use of the nutrition facts panel and awareness federal nutrition programs and guidelines among US adolescents, with particular attention to differences in population groups based on race/ethnicity. We secondarily evaluated the relationship between awareness and frequency of use and risk for overweight and obesity.

## Methods

The NHANES 2005–6 survey incorporated new questions to evaluate nutrition awareness of the Dietary Guidelines for Americans, the Food Guide Pyramid, the 5-A-Day Program, and diet behavior such as use of the nutrition facts label and the ingredient list. The NHANES is a continuous national survey that represents a stratified multistage probability sample of the non-institutionalized US population. Health interviews are conducted in participant’s homes and measurements are performed in specially designed and equipped mobile centers [12]. The interview team consists of a dietary and health interviewers and a physician. Most of the staff are bilingual in English and Spanish. An advanced computer system is used to enter respondents’ information using notebook computers. Respondents are able to enter their own responses to sensitive questions in privacy using a touch-sensitive computer screen [12]. Transportation to and from the mobile center is provided if necessary and participants are provided with compensation for participation. Adolescents 16 and 17 years of age have to have a parent read and sign the consent form in addition to signing a Household Interview Consent Form [13].

This study investigated the relationship between responses to the diet behavior questions using NHANES 2005-6 data for adolescents between the ages of 16 and 19 (n = 1160 designed to represent a sample size of 16,204,982). Seventeen different questions were asked on nutrition awareness and diet behavior as part of the NHANES 2005–6, although only a sub-sample of the 1160 answered questions on label reading behavior as described below. The main outcomes of interest were (1) awareness of nutrition program, and (2) active use of nutrition panel information defined as ‘always' or ‘most of the time’ using the nutrition fact label information.

Specifically, awareness of nutrition guidance programs such as the Dietary Guidelines for Americans, the Food Guide Pyramid and the 5-A-Day for Better Health Program was assessed (3 questions) as was use of the nutrition facts panel and additional label reading behavior (ingredient list, serving size or health claims information) (4 questions). The questions were of the form, “Have you heard of The Dietary Guidelines (or other nutrition program)?” and was repeated in the same format for the other nutrition guidance programs [14]. Participants could answer ‘yes’ or ‘no’ that they were aware of one of the nutrition programs.

For the question on the food label they were given a card with a food label on it and asked, “Here is an example of a food label. This part of the food label is called the “Nutrition Facts” panel. How often do you use the Nutrition Facts panel (or other part of the food label) when deciding to buy a food product?” [14] They could answer that they ‘never’, ‘rarely’, ‘sometimes’, ‘most of the time’ or ‘always’ made use of the nutrition facts panel information, ingredient list, serving size or health claims information. As part of the NHANES dietary behavior interview process, additional label reading behavior was assessed among the sub-sample of adolescent respondents who made use of the nutrition facts panel, the serving size information, the ingredient list or the health claims on the package (n = 742). In this subgroup, label reading behavior was assessed for use of total calories, calories from fat, total fat content, trans fat content, saturated fat, cholesterol, sodium, carbohydrates, fiber and sugars in making a product selection (10 questions). The questions were phrased in the survey as follows, “When you use the food label to decide about a food product, how often do you look for information about X? [14] Would you say always, most of the time, sometimes, rarely or never?”

Quality assurance and control measures included using trained interviewers with a Computer-Assisted Personal Interviewing (CAPI) system to interview each participant. The CAPI system has built-in consistency checks to reduce data entry errors. All data were reviewed by NHANES field staff for completeness and accuracy [15]. NHANES documentation does not indicate that the diet behavior questions which specifically asked about awareness of federal nutrition programs and use of nutrition facts panel information were tested for validity or reliability.

As part of the NHANES protocol, participants were weighed and measured. To collect weight measurements, participants stood on a floor scale, equipped with a digital read-out. Standing height was measured using a wall-mounted stadiometer. The stadiometer was connected to an automated data electronic database and data were entered automatically [16]. Body mass index (BMI) was calculated as weight (kg)/(height (m)) [2] and CDC growth charts and classifications were were used to determine overweight and obesity [17]. Adolescent overweight was defined as having a BMI percentile ≥85^th^ or, for those 18 years or older, a BMI ≥25 and <30. Adolescent obesity was defined as having a BMI percentile ≥ 95th or a BMI ≥ 30.

Percentages and 95% confidence intervals were calculated for all means and frequencies. We applied chi-squared tests to evaluate differences in proportions and student’s t-tests to evaluate differences in means to compare how socio-demographic and health variables differed in adolescents who were aware of nutrition programs and made active use of the nutrition panel information in comparison with those with did not. Unadjusted logistic regression models were used to assess binary outcomes for awareness of nutrition programs and frequent use of the nutrition facts label (always or most of the time) in relation to adolescent BMI percentile or category. Interaction terms for race/ethnicity and different nutrition label reading behaviors and nutrition awareness were evaluated to determine possible interactions between these variables and risk for overweight or obesity. Multivariate logistic models were used to compute adjusted estimates for the relation between nutrition awareness and label reading behavior and risk for adolescent overweight. Models were adjusted for adolescent age, race/ethnicity, foreign born versus US born and living in poverty. NHANES supplied sampling weights and strata were used to analyze the population survey data. All analyses were done using Stata 11.0 using svy commands for survey data. Data are expressed as means ± standard errors (SE).

The NHANES 2005–6 surveys received approval from the National Center for Health Statistics Ethics Review Board (ERB) (Protocol # 2005–6) [15]. The University of California, San Francisco (UCSF) Committee on Research (CHR) concluded that the study did not need ethical approval as all analyses involved de-identified data.

## Results

The mean age of the adolescents surveyed was 17.5 ± 1.1 years. Most were non-Hispanic white (62.8%), with sizable representation of non-Hispanic African-Americans (15.8%) and Mexican-Americans (15.8%). The sample was largely born in US (90.4%), with 20.0% living in poverty (Table 1).

**Table 1:** Sociodemographics of national health and nutrition examination survey (NHANES)

Adolescent Sample (n=1160)	
Variable	Mean ± SD or % (CI)
Demographics	
Age (years)	17.5 ± 1.1
Race/Ethnicity	
Non-Hispanic white	62.8 (54.4-705)
Non-Hispanic black	15.8 (9.7-24.5)
Mexican-American	10.7 (7.8-14.4)
Other Hispanic	4.9 (3.3-7.2)
Other	5.9 (3.3-7.2)
Poverty to Income Ratio (PIR)	2.62 (2.4-2.84)
US Born	90.4 (87.4-92.7)
Living in Poverty (PIR < 1.3)	20.0 (15.3-25.7)

Almost all of the adolescents surveyed were aware of the Food Guide Pyramid (92.4%, 95%CI 89.8-94.4). Fewer (29.3%, 95%CI 25.1-33.8) were aware of the USDA’s Dietary Guidelines for Americans, while 43.5% (95%CI 38.1-49.6) were aware of the Center for Disease Control’s (CDC) 5 A Day for Health Program (Table 2). Less than 25% of adolescents surveyed stated that they regularly (always or most of the time) made use of the information on the nutrition facts label, with the highest percentage using the total fat on the nutrition facts label (Table 2). Close to 25% stated that they regularly checked calories from fat on the nutrition facts label. Less than 15%, stated that they regularly checked the cholesterol on the nutrition facts label, looked at the ingredient list, used serving size or health claims, or checked sodium or fiber on the nutrition facts label (Table 2). For all questions asked concerning the nutrition facts label, with the exception of checking calories and total fat, more than 50% of adolescents stated that they rarely or never checked the nutrition information.

**Table 2:** Nutrition awareness and label reading behaviors in Us adolescents

Variable	Yes or Always % (CI)	Sometimes % (CI)	No or Rarely/Never % (CI)
Awareness of Federal Nutrition Programs (n=1160)			
Heard of Dietary Guidelines	29.3 (25.1-33.8)		70.7 (66.2-74.9)
Heard of Food Pyramid	92.4 (89.8-94.4)		7.6 (5.6-10.3)
Heard of 5-a-day Health Program	43.5 (38.1-49.6)		56.2 (50.4-61.9)
Nutrition Label Reading Behaviors (n=1160)			
Use Nutrition Facts Panel	16.7 (13.4-20.7)	20.4 (16.5-24.9)	62.4 (58.7-65.9)
Use Ingredient List on Food Label	9.0 (6.4-12.1)	16.1 (13.0-19.7)	74.5 (70.2-78.4)
Use Serving Size on Food Label	11.2 (8.9-14.0)	19.7 (16.4-23.5)	68.8 (65.4-72.0)
Use Health Claims on Food Package	9.3 (6.0-14.0)	18.2 (14.5-22.6)	71.8 (67.2-76.1)
Specific Nutrition Facts Reading Behaviors (n=742)			
Check Calories on Food Label	16.0 (13.5-18.8)	26.5 (22.7-30.7)	49.1 (43.9-54.4)
Check Calories from Fat on Food Label	23.7 (18.4-29.9)	25.4 (22.0-29.0)	50.1 (45.6-56.4)
Check Total Fat on Food Labels	26.5 (21.8-31.7)	24.2 (19.1-30.3)	49.3 (43.5-55.2)
Check Trans Fat on Food Labels	16.4 (11.6-22.6)	20.5 (16.5-25.2)	63.2 (56.8-69.1)
Check Saturated Fat on Food Labels	17.9 (14.1-22.4)	22.9 (18.5-27.9)	59.2 (51.6-66.4)
Check Cholesterol on Food Labels	13.0 (9.0-18.3)	26.6 (22.4-31.3)	60.4 (54.0-66.5)
Check Sodium on Food Labels	13.3 (10.4-17.0)	20.4 (17.4-23.7)	66.3 (61.6-70.7)
Check Carbohydrates on Food Labels	21.8 (18.0-26.1)	25.8 (20.7-31.5)	52.5 (47.0-57.9)
Check Fiber on Food Labels	10.8 (9.0-13.3)	23.6 (19.7-28.0)	65.6 (61.5-69.6)
Check Sugars on Food Labels	21.6 (16.4-27.9)	26.3 (22.4-30.7)	52.1 (44.6-59.5)

Significant differences in frequency based on race/ethnicity were found for all awareness of nutrition programs and use of nutrition facts label among those surveyed. Non-Hispanic whites had the highest frequency of awareness of the Food Guide Pyramid and 5-A-Day Health Program, while other Hispanics had the highest awareness of Dietary Guidelines for Americans (Table 3). Mexican Americans had the lowest awareness of all three programs.

**Table 3:** Nutrition awareness and label reading behaviors in adolescents by race and ethnicity

	Mexican-American % (CI)	Other Hispanic % (CI)	African-American % (CI)	White % (CI)
Variable				
Nutrition Awareness* (n=1160)				
Heard of Dietary Guidelines^	11.9 (9.9-14.3)	41.1 (19.3-67.0)	29.1 (19.9-40.3)	31.0 (20.4-44.0)
Heard of Food Pyramid^	75.6 (68.6-81.5)	89.7 (63.6-97.7)	89.7 (83.5-93.7)	96.5 (92.2-98.8)
Heard of 5-a-day Health	30.2 (23.3-38.2)	41.3 (19.1-67.7)	44.5 (37.6-51.6)	45.2 (36.5-54.2)
Label Reading Behaviors^1^(n=1160)				
Use Nutrition Facts	14.2 (9.6-20.7)	13.0 (4.4-32.7)	12.0 (8.3-16.9)	18.3 (13.5-24.3)
Use Ingredient List	7.1 (4.3-11.5)	13.0 (3.2-40.6)	8.3 (5.7-11.8)	9.2 (5.8-14.3)
Use Serving Size	9.7 (6.4-14.3)	6.4 (0.7-38.5)	8.0 (6.0-10.5)	12.3 (8.8-16.9)
Use Health Claims^	7.9 (4.5-13.5)	6.0 (1.1-27.0)	5.2 (3.5-7.7)	9.6 (5.3-16.7)
Specific Nutrition Facts Behaviors (n=742)				
Check Calories	14.9 (10.1-21.4)	14.5 (4.0-40.7)	19.8 (10.9-33.2)	17.8 (14.3-21.8)
Check Calories from Fat	21.8 (16.3-28.4)	17.6 (7.7-35.1)	16.1 (13.0-19.8)	25.2 (18.3-33.7)
Check Total Fat	23.6 (17.8-30.5)	36.2 (18.3-59.1)	16.9 (14.3-19.8)	27.9 (21.4-35.5)
Check Trans Fat^	14.7 (11.4-18.7)	2.4 (1.3-4.1)	10.0 (6.7-14.6)	18.7 (12.1-27.6)
Check Saturated Fat	13.7 (9.6-19.1)	16.9 (4.5-4.7)	11.4 (8.7-14.8)	20.1 (14.3-27.5)
Check Cholesterol	17.4 (14.2-21.1)	8.7 (1.1-46.1)	15.5 (12.8-18.5)	12.7 (7.3-20.9)
Check Sodium	10.5 (7.6-14.3)	15.8 (3.9-46.7)	14.7 (10.8-19.8)	13.5 (10.0-17.9)
Check Carbohydrates	19.8 (15.1-25.5)	29.1 (11.9-55.6)	12.2 (9.2-16.0)	24.6 (20.1-29.6)
Check Fiber	10.0 (6.8-14.4)	14.5 (3.4-44.)	8.7 (6.4-11.7)	10.9 (8.0-14.6)
Check Sugars	21.4 (16.5-27.3)	23.2 (7.8-51.9)	19.1 (14.7-24.4)	22.3 (15.1-31.5)

*Participant answered yes.

^Significant at p<0.05.

^1^Participant answered always or most of the time.

Race and ethnic background were associated with significant differences in nutrition awareness and label reading behaviors. Non-Hispanic white adolescents were more likely to check calories from fat, trans fat, saturated fats, the serving size information, and the nutrition facts label (Table 3). Other Hispanics were more likely to check sugars, fiber, and carbohydrates, sodium and the ingredient list (Table 3). Non-Hispanic African-Americans were least likely to check any of the fat information (calories from fat, total fat, trans fats and saturated fat) compared with the other racial/ethnic groups (Table 3). Statistically significant differences between ethnic/racial groups were found for awareness of the Food Pyramid, the use of health claims on the food package and checking trans fats (Table 3).

Few label-reading behaviors were associated with increased risk for overweight and obesity. Checking cholesterol on the food label was associated with increased risk for overweight and obesity but only in unadjusted analysis (OR 1.89; 95%CI 1.02-3.50) (Table 4). Other label reading behaviors were not associated with risk for overweight or obesity (Table 4). Evaluating the relationship between awareness of nutrition programs, reading nutrition facts labels and overweight and obesity in adolescents, only having heard of the 5-A-Day Health program was associated with decreased risk for obesity in adjusted analysis (OR 0.65; 95%CI 0.46-0.96) and unadjusted analysis (OR 0.66; 95%CI 0.46-0.96) (results not shown). For overweight and obesity, having heard of the 5-A-Day Health Program neared significance in unadjusted (OR 0.83; 95%CI 0.66-1.04) and adjusted (OR 0.82; 95% 0.66-1.02). There was little difference between the unadjusted and adjusted odds ratios evaluating the relationship between awareness of nutrition programs, use of nutrition information and risk for overweight and obesity, except for the loss of significance in the relationship between check cholesterol on food labels and overweight and obesity in adjusted analysis (Table 4). Of note, of the 17 nutrition variables evaluated in relationship to risk for overweight and obesity in multivariate analysis, not one was significant. In the analysis of interaction between race/ethnicity, overweight and obesity and label reading behaviors, those who were Mexican-Americans, in comparison with whites, and used the nutrition facts panel information or checked trans fats on the nutrition facts label were much more likely to be overweight or obese (OR 3.13; 95%CI 1.56-6.35; OR 2.50; 95% CI 1.08-5.80 respectively).

**Table 4:** **Relationship Between BMI≥85**^**th**^**percentile or BMI≥25 in Adolescents and Nutrition Awareness and Label Reading Behaviors**

Variable	Odds Ratio (OR) 95%	Adjusted^2^ OR 95%CI
Nutrition Awareness ^3^(n=1160)	Confidence Interval (CI)^1^	
Heard of Dietary Guidelines	1.04 (0.81-1.34)	1.06 (0.81-1.40)
Heard of Food Pyramid	0.72 (0.40-1.27)	0.72 (0.37-1.40)
Heard of 5-a-day Health Program	0.83 (0.66-1.04)	0.82 (0.66-1.02)
Nutrition Label Reading Behaviors^4^ (n=1160)		
Use Nutrition Facts Panel	0.80 (0.45-1.40)	0.82 (0.66-1.02)
Use Ingredient List on Food Label	1.30 (0.83-2.04)	1.40 (0.90-2.19)
Use Serving Size on Food Label	1.03 (0.50-2.15)	1.06 (0.50-2.22)
Use Health Claims on Food Package	0.93 (0.58-1.47)	1.02 (0.66-1.57)
Specific Nutrition Facts Reading Behaviors (n=742)		
Check Calories on Food Label	1.48 (0.95-2.31)	1.61 (1.03-2.51)
Check Calories from Fat on Food Label	1.23 (0.76-1.99)	1.36 (0.81-2.27)
Check Total Fat on Food Labels	1.00 (0.57-1.77)	1.09 (0.61-1.97)
Check Trans Fat on Food Labels	0.97 (0.58-1.62)	1.03 (0.59-1.79)
Check Saturated Fat on Food Labels	1.10 (0.62-1.95)	1.18 (0.66-2.14)
Check Cholesterol on Food Labels	1.89 (1.02-3.50) *	1.76 (0.95-3.24)
Check Sodium on Food Labels	1.57 (0.85-2.92)	1.59 (0.86-2.94)
Check Carbohydrates on Food Labels	1.26 (0.74-2.14)	1.32 (0.80-2.16)
Check Fiber on Food Labels	1.00 (0.57-1.76)	1.04 (0.61-1.78)
Check Sugars on Food Labels	1.26 (0.81-1.95)	1.29 (0.82-2.04)

^1^Unadjusted logistic regression models.

^2^Multivariate logistic regression models. Odds ratios are adjusted for age, race/ethnicity, foreign born versus US born and poverty status.

^3^Participant answered yes.

^4^Participant answered always or most of the time *P<0.05.

## Discussion

Our study is the first to detail the frequency of nutrition facts label use among adolescents using a population-based survey, and the first to evaluate association between nutrition facts label use and overweight and obesity. A low percentage of US adolescents regularly use the information on the nutrition facts label, with more than 50% rarely or never using the nutrition information. Similarly, a low percentage were familiar with the Dietary Guidelines for Americans, but almost half were aware of the 5-A-Day program and almost all had heard of the Food Guide Pyramid.

Our results contrast with previous studies that have found a higher percentage of US adolescents reading the nutrition facts label [18-20]. However, none of these studies was population-based, and the adolescents/young adults were older than those surveyed in our study. Our results are also much lower than previous population-based surveys with adults. The 1995, 1997 and 2010 Shopping for Health surveys found between 54% and 68% of US adult consumers regularly use nutrition labels when shopping for food items [21-23].

This is the first study to suggest significant racial/ethnic differences in awareness of nutrition programs and use of the information on the nutrition labels. Specifically, Mexican-American adolescents had the lowest awareness of nutrition programs, and African-American adolescents were the least likely to check fat information on the nutrition facts label. This differs sharply from other studies with adults that have found 78% of African-Americans read nutrition labels when purchasing food items [24]. Our results may have diverged from previous studies with older African-American due to concomitant health issues such as diabetes mellitus or hypertension that necessitate more frequent label reading, in contrast with the younger population of African-American adolescents surveyed in NHANES who likely do not have a high prevalence of these health issues. Other studies have found that adults with chronic disease have a higher frequency of use of nutrition facts labels [25].

Recently, Wright and Wang [26] analyzed awareness of federal dietary programs in adults older than 15 years of age using the same NHANES 2005–6 survey data. They found that that awareness of the Dietary Guidelines for Americans was lowest among older adolescents 16–19 years and higher in older age groups, while the awareness of the Food Guide Pyramid was highest among adolescents compared with older age groups [26]. Knowledge of the 5-A-Day Program was lower among adolescents compared with adults < 60 years but comparable with adults ≥60 years of age. Similar to our results of low awareness among Mexican-Americans of federal dietary programs, Wright and Wang found the lowest awareness of federal dietary guidance among Mexican-Americans in all age groups [26].

As significant racial and ethnic differences persist in the incidence of childhood obesity [25], intervention programs should attempt to address these disparities. Non-governmental organizations and governmental task forces are pushing the Obama administration and the Food and Drug Administration (FDA) to adopt more consumer friendly nutrition facts labels in part to address the obesity epidemic [11]. As the US government debates how to best address the obesity epidemic and how to provide accessible nutrition information to Americans, it is imperative to recognize the current low use by US adolescents and especially the lowest use among non-Hispanic African-American and Mexican-American adolescents.

Of concern, we also found that a low percentage of US adolescents were aware of the USDA’s Dietary Guidelines for Americans, and less than half were aware of the 5-A-Day for Better Health Programs. One of the recommendations from the 2010’s White House Task Force on Obesity was to disseminate important nutrition information from the 2010 Dietary Guidelines through simple, easy actionable messages [11]. However, educators and public health workers should be aware of the low reach of the current guidelines among adolescents. This may be particularly important as we found that awareness of the 5-A-Day for Better Health Program was associated with reduced risk for obesity. As a diet high in fruits and vegetables has been associated with reduced risk for obesity [27], we suggest expanding this CDC program to all children and adolescents and potentially incorporating aspects into the expanded nutrition education programs promoted by the Let’s Move campaign for school-based nutrition education [11]. Previous studies have also found that providing nutrition educational information on fruits and vegetables and computer based communications can increase fruit and vegetable intakes in adolescents and young adults, suggesting that a more targeted intervention using the 5-A-Day program could have a positive impact [28].

Of note, none of the 17 nutrition-awareness and label reading variables evaluated in relationship to overweight and obesity were significant in multivariate analysis. It is possible that this lack of association may be related to the overall low prevalence of label reading behavior among adolescents, in general, for many of the behaviors surveyed. Further studies need to evaluate why there was no association between awareness of nutrition programs and overweight and obesity in adolescents. It is possible that while many of the adolescents were aware of the programs, they were not knowledgeable of the components of the programs or the guidance recommendations.

### Limitations

There were specific limitations in conducting this study that should be considered in interpreting the results. As questions on awareness of nutrition programs and frequency of use of nutrition information were included only in the 2005–6 NHANES and not other cycles of the survey, the sample size for this study was relatively small. It would have been preferable to use two or more cycles of the NHANES survey. Additionally, as we conducted many statistical tests to assess for possible association between awareness of nutritional programs and use of nutrition facts panel information, some of our positive results could have been the result of chance given the number of hypotheses that were tested. Further studies need to validate our findings with other adolescent groups.

Also, while the NHANES set of surveys does collect information on dietary intake including information on detailed macro and micronutrient intake, we did not evaluate awareness of federal nutrition program or use of nutrition information in relation to adolescent dietary intake. As the primary goal of this study was to quantify awareness of nutrition programs and use of nutrition facts panel in adolescents as well as assess risk in relation to obesity and overweight, the relationship between these factors and dietary intake was outside the purview of this study.

## Conclusions

Previous studies with adults and adolescents have found that use of the nutrition facts panel is associated with reduction in overall energy and fat intake [1,2], although studies have not looked at the relationship between use of labels or nutrition awareness and risk for overweight and obesity. Alternatively, our finding an absence of association may suggest that awareness of nutrition programs and use of nutrition labels may not be a sufficient intervention or strategy alone to reduce overweight and obesity in adolescents. We did find some race/ethnic specific differences in relation to use of nutrition labels and race/ethnicity. Specifically for Mexican-American adolescents, use of the nutrition facts label and checking the amounts of trans fats in a product was associated with increased risk for overweight and obesity. Previous studies with adults have also found that obesity is associated with increased attention to certain parts of the nutrition facts label and likely reflects concern about losing weight or underlying health conditions [29].

Follow-up studies, however, should assess the relationship between use of labels and awareness of programs and meeting certain nutritional standards such as the dietary reference intake (DRI) for each nutrient.

For follow-up studies, we also recommend collecting qualitative information from adolescents to ascertain what aspects of the nutrition facts label might be unclear and to better understand why the use of nutrition facts labels is relatively low in young Americans. For example, limited provision of nutrition or health education in many US schools may lead to lack of awareness on the importance of reading labels, thus leading to less informed dietary decisions. Alternatively, adolescents may experience apathy and disinterest in spite of adequate nutrition education and knowledge about the healthy eating practices; qualitative studies will help untangle the reasons for the low adolescent use of nutrition information.

## Competing interests

The authors declare that they have no competing interests.

## Authors’ contributions

JW and MH conceived of the study. JW conducted the statistical analyses and interpretations. JW and MB wrote up the results and approved the final manuscript.

## Pre-publication history

The pre-publication history for this paper can be accessed here:

http://www.biomedcentral.com/1471-2431/12/55/prepub
